# An efficient electricity theft detection based on deep learning

**DOI:** 10.1038/s41598-025-93140-z

**Published:** 2025-04-15

**Authors:** Nada M. Elshennawy, Dina M. Ibrahim, Ahmed M. Gab Allah

**Affiliations:** 1 Department of Computers and Control Engineering, Faculty of Engineering, Tanta University, Tanta, 31733 Egypt; 2 Department of Information Technology, College of Computer, Qassim University, 51452 Buraydah, Saudi Arabia; 3 Department of Information Systems, Faculty of Computers and Artificial Intelligence, University of Sadat City, Sadat City, Monufia 32897 Egypt

**Keywords:** Electricity theft detection, Smart grids, LoRAS, Convolutional neural networks (CNN), Long-short term memory (LSTM), Energy science and technology, Renewable energy

## Abstract

Electrical theft is a pervasive issue that has detrimental impacts on both utility companies and electrical consumers worldwide. It undermines the economic growth of utility businesses, poses electrical risks, and affects customers’ expensive energy bills. Smart grids produce vast quantities of data, including consumer usage data which is crucial for identifying instances of energy theft. Machine learning and deep learning algorithms may use this data to identify instances of energy theft. This research presents a new approach using convolutional neural networks and long-short-term memory to extract abstract characteristics from power consumption data, to improve the accuracy of theft detection for electricity users. We mitigate dataset shortcomings, such as incomplete data and imbalanced class distribution, by using LoRAS data augmentation. The method’s efficiency is evaluated by using authentic power usage data obtained from the State Grid Corporation of China. Finally, we demonstrate the competitiveness of our approach when compared to other approaches that have been assessed on the same dataset. During the validation process, we attained a 97% accuracy rate, surpassing the highest accuracy reported in previous studies by 1%. We obtained accuracy values of 98.75%, 95.45%, and 97.7%, along with corresponding recall and F1 scores. The findings indicate that the suggested approach surpasses existing state-of-arts methods.

## Introduction

Electricity plays an essential part in human existence, and almost every individual on the planet can use it. Considering its extensive worldwide use, we must tackle concerns such as electrical power wastage^1,2^. The distribution system operators categorize electricity loss into technical loss (TL) and nontechnical loss (NTL) based on their opinions^3^. TL refer to the dissipation of electrical energy inside the power distribution network caused by the thermal effects and combustion of hardware components used for electricity transmission, including electric transformers, cables, conductors, and other equipment. NTL represents the quantity of power that is used but not accounted for in billing. The occurrence of this phenomenon may be attributed to a multitude of circumstances, such as measurement inaccuracies, faulty smart meters (SMs), electrical pilferage, and other similar causes^4^.

As field inspections improve and smart grids (SGs) become more common, old ways of stealing electricity, such as meter bypassing, become less useful. New methods of stealing electricity, such as SMs transmission, have taken their place. One can tamper with the SM readings before, within, and after the SMs^1^. NTL detection uses traditional theft detection methods. However, both traditional game theory and state-based methods are not optimal and costly, respectively^5^. Currently, advanced metering infrastructure (AMI) collects a vast amount of historical electricity consumption (EC) data from electricity consumers, making machine learning (ML) techniques easily available^6–15^. As a result, these techniques achieve satisfactory and improved performance for electricity theft detection (ETD) in SGs. The ML techniques use consumer EC histories collected by the AMI in SGs.

Normally, electricity consumers’ EC adopts a specific symmetric and synchronized statistical pattern. On the other hand, asymmetry in consumers’ EC profiles can serve as a sign of theft or abnormal activity. We train the ML classifier on the EC history database to predict and identify malicious patterns. Since these classifiers utilize the ready-made SM-EC history, their costs are not high. However, there are some problems with the available ML-based theft classification techniques, which restrict their performance concerning theft classification metrics. One of the problems is class imbalance^16^. This problem pertains to the disparity in the number of electricity theft and nontheft users. Moreover, nontheft users heavily outnumber theft users. It is due to the normal phenomenon that the EC instances for nontheft users are available in a huge range, while the theft samples are very rarely available or sometimes do not exist in the real environment. The curse of dimensionality is another issue with employing ML classifiers in ETD tasks. This aspect refers to the issue that arises while working with high-dimensional data. In such situations, classification loss is directly proportional to the number of features (dimensions). Ignoring this issue hurts the ML classifiers’ performance in terms of theft classification metrics^3^. Another crucial issue is ignoring the automated tuning of hyperparameters in the ML classifiers. Manual tuning of hyperparameters affects the ML algorithm’s performance in terms of computational overhead and classification metrics^17^.

The extent of progress in various technological and research fields can be demonstrated through a set of researches^18–20^. Several papers highlight innovative optimization techniques applied to IoT and industrial IoT (IIoT) environments. For instance,^21^ leverages the Grey Wolf optimization algorithm with MapReduce to optimize quality of service in IoT service composition, while^22^ employs Glowworm Swarm Optimization to enhance blockchain-based IIoT systems. Similarly,^23^ integrates an artificial bee colony algorithm with genetic operators to improve data aggregation reliability in IIoT settings, demonstrating advancements in scalability, reliability, and energy efficiency. IoT healthcare services also benefit from nature-inspired algorithms^24^ and deep learning techniques^25–27^, which address challenges like adaptability, scalability, and real-time data processing for applications ranging from diagnosis to drug discovery.

Emerging technologies like blockchain and federated learning^28,29^ are central to addressing security and privacy challenges, particularly in combating deepfake proliferation and enhancing drone security in IoT frameworks. Artificial intelligence methods are also being applied to climate change^30^, conversational AI^31^, and astrophysical numerical computations^32^. These works emphasize the transformative impact of AI and machine learning across various fields, from environmental sustainability to real-time conversational models. Additionally, historical insights into computing^33^ and the evolution of societal technological waves toward IoT^34^ provide contextual understanding of how these advancements fit within broader historical and technological paradigms.

Finally, challenges in specialized areas, such as botnets^35^, vehicular ad hoc networks^36,37^, and Alzheimer’s disease^38^, are addressed with innovative solutions. These include novel intrusion detection systems, fuzzy multicriteria decision-making approaches for mitigating network congestion, and deep learning applications for healthcare, all aimed at improving security, efficiency, and healthcare outcomes. Together, these studies underscore the importance of interdisciplinary innovation in addressing complex technological and societal challenges.

Since classification approaches usually need large volumes of labelled historical power consumption data, several machine learning models are used for electricity theft detection. Convolutional neural networks (CNNs)^39^, support vector machines (SVM)^15^, and other artificial neural networks^40,41^ are a few examples. Unsupervised techniques, on the other hand, like clustering, concentrate on the data that lacks labels. For instance,^42^ computed users’ anomaly degrees using density-based spatial clustering of applications with noise (DBSCAN). Various solutions for detecting NTL have been introduced to address the increasing problem of power fraud and theft detection. The approaches may be classified into hardware-based methods, statistical and game theory methods, and data-driven methods.

Hardware-based methods, Hardware solutions depend on devices to monitor grid system metrics such as energy, current, and voltage^43^. The main disadvantage of this strategy is that it necessitates the acquisition of costly new equipment. The use of sophisticated metering infrastructure in the SG has enabled the acquisition of a substantial volume of data. As a result, data-driven strategies for recognizing NTL are becoming more prevalent^44^. Therefore, the majority of ideas in the literature prioritize data-driven approaches above hardware-based solutions^5^. Compared to other hardware options, these techniques are less complicated and cheaper to implement.

Methods based on statistical and game theory, The fundamental basis for game theory-based approaches to identify the NTL is establishing a game between the service provider and deceitful clients^5^. Several power theft detectors have been developed utilizing game theory^44–46^ data mining, and statistical approaches such as state estimation^47^, clustering, local outlier factor (LOF), and principal component analysis (PCA). For example,^48^ used the k-means clustering technique to cluster clients based on their power usage measurements. Outlier candidates with substantially differing consumption measurements from the centre of their clusters were identified using LOF. In addition, LOF is used to calculate the anomaly score for each of the detected outlier candidates. Furthermore, Singh et al.^49^ suggest a PCA-based detector. Zheng et al.^50^ tackle the issue by combining two data mining methodologies. The maximum information coefficient (MIC) approach identifies relationships between NTL and consumer EC. Another method is the clustering approach known as fast search and discovery of density peaks (CFSFDP). Nevertheless, statistical and game theory approaches lack precision since they are unable to capture the temporal aspect and intricate patterns of the data^51^.

Data-driven methods and artificial intelligence (AI) algorithms play a crucial role in optimizing energy use in the energy industry. AI algorithms can autonomously monitor and analyze consumers’ energy use trends. It may be possible to identify incidents of power theft with accuracy by looking through the data that smart meters gather^52^. Many ML-based detectors have been suggested in the literature to identify fake power consumption measurements that malicious SMs report.

El-Toukhy et al.^6^ develop a deep reinforcement learning (DRL) and apply it to the Irish smart energy trails (ISET) dataset^53^. The DRL is outlined in four distinct scenarios. The initial model employs either a single or double deep Q network (DQN) to calculate the value of the Q function using state and action as inputs. This model is used to detect cases of power theft and cyber-attacks. Second, to achieve high detection accuracy and prevent zero-day attacks, the global detector is used to create a tailored detection model for new clients. Furthermore, the third scenario involves considering the alteration of the consumption behaviour among the current clients. The fourth scenario focuses on addressing the issues of protecting against recently initiated cyber-attacks. Another work uses a two-stage deep-learning-based algorithm for identifying energy theft using the ISET dataset using a DL architecture that can represent the suspicious actions of dishonest clients using k-means clustering. The model achieves an accuracy of 98.1%. Similarly, Elgarhy et al.^54^ examine the susceptibility of DNN-based power theft detectors to evasion assaults and the influence of the model’s regularization (generalization) on its resilience. We categorize the highly proficient users into clusters, develop a detector for each cluster, and evaluate the efficiency and robustness of this detector compared to a global detector that is trained on data from all consumers. Shehzad et al.^12^ suggested a structure with two phases. In phase one, the ML classifiers extract a wide range of knowledge and acquire new information using data on power use. Phase 2 utilizes an MLP as a meta-learner, using the prediction outputs of the classifiers as input. To classify both benign and malevolent activities. The Pakistan Residential Electricity Consumption (PREC) dataset was used to obtain a model with ROC-AUC and PR-AUC values of 0.910 and 0.988, respectively.

On the other hand, Lepolesa et al.^8^ examined the detection of power theft in SGs using time-domain and frequency-domain information in a DNN-based classification technique. Next, improve the accuracy of identifying power theft by fine-tuning the parameters using a Bayesian optimizer and including an adaptive moment estimation optimizer. Perform experiments using different values for critical parameters to determine the ideal settings that provide the maximum level of accuracy. The model is tested on a public dataset^55^ and achieve the best accuracy of 91.8%. Pamir et al.^9^ proposed a model called the salp swarm algorithm (SSA), gate convolutional autoencoder (GCAE), and cost-sensitive learning and long short-term memory (CSLSTM), an effective ETD model named SSA–GCAE–CSLSTM. The CSanSTM model is used for theft classification, the hyperparameters of the proposed model are tuned automatically using an SSA, and GCAE is used to extract the distinctive and significant characteristics while avoiding the problem of the curse of dimensionality. The SSA–GCAE–CSLSTM model achieves an accuracy of 92.25%.

Other approaches using RNN models such as the DFL-ConvGRU model^10^ train a Convolutional Gated Recurrent Unit (ConvGRU) model using distributed data sources without violating data privacy by utilizing the collaborative capabilities of deep federated learning. The Open Energy Data Initiative (OEDI) portal^56^ is used by the DFL-ConvGRU model and achieves 97.84% accuracy. Huang et al.^11^ use temporal convolutional networks (TCN) as a component to extract daily-scale aspects of power consumption. To handle long-term dependencies in time series data, they also use long short-term memory (LSTM). The authors use a multi-level feature extraction module called LSTM-TCN and a deep convolutional neural network (DCNN) to simultaneously extract features at different sizes. Subsequently, the authors integrate and feed the retrieved information into a fully connected (FC) layer for classification, enabling accurate identification of compromised users. The suggested approach has an impressive detection accuracy of up to 94.7% applied to the State Grid Corporation of China (SGCC) dataset^39^[18].

Furthermore, Ibrahim et al.^57,58^ a novel methodology, known as STDL, for gathering power consumption data in advanced metering infrastructure networks while safeguarding the privacy of users. The authors accomplished this by using a deep-learning technique to either transmit deceptive signals or duplicate genuine ones. The authors first use K-means clustering and actual power consumption measurements to create a dataset^59^ for transmission patterns, and our assessments reveal that the success rate of the attacker stands at about 91%. Ultimately, we use a defensive mechanism based on deep learning to successfully counteract the presence-privacy assault by efficiently transmitting spoofing signals.

Based on research findings^13–16,60–63^, it is possible to enhance the efficacy of energy theft detection by using various ML techniques. For illustration, Kawoosa et al.^13^ used principal component analysis (PCA) to reduce the size of the dataset and an XGBoost-based detector to find electricity theft. The State Grid Corporation of China (SGCC) used the tested dataset^62^. The results indicate that both the detection rate and the false positive rate (FPR) achieved 96%. Random forest, XGBoost, and multi-layer perceptron ML classifiers are aggregated together and return the results using a weighted majority approach. This model is applied to a public dataset^63^ achieving the best accuracy in the range of 88%–94.70%^16^. A metered data theft detector based on XGBoost is presented in^14^. In Jindal et al.^15^ provide a thorough top-down approach that utilizes a decision tree (DT) and support vector machine (SVM). Based on experimental findings, this approach is feasible enough to be implemented in real time and decreases false alarms greatly. Table 1 summarizing the pros and cons of the techniques used in the listed studies while Table 2 provides a summary of the algorithms, data sources, and limitations of the associated research.

**Table 1:** The pros and cons of the techniques used in the listed studies.

Study	Pros	Cons
El-Toukhy et al. ^6^	- Handles dynamic and evolving electricity theft patterns. - Learns optimal actions through reward-based training. - Scalable for large smart grid systems.	- High computational requirements for training. - Requires fine-tuning for convergence. - Black-box nature limits interpretability.
Emadaleslami et al. ^7^	- Improved accuracy by separating detection stages. - Optimized for Advanced Metering Infrastructure environments. - Reduces false positives in theft detection.	- Dependent on quality of input features. - Increased system complexity due to multi-stage approach. - High reliance on labeled data.
Lepolesa et al. ^8^	- High detection accuracy for theft patterns. - Suitable for large datasets. - Capable of processing non-linear relationships in data.	- Computationally expensive for large-scale applications. - Requires extensive hyperparameter tuning. - Susceptible to overfitting on imbalanced datasets.
Pamir et al. ^9^	- Effective in optimizing energy consumption. - Capable of analyzing complex theft patterns. - High scalability for diverse grid environments.	- High training and inference costs. - Requires large labeled datasets for effectiveness. - Black-box nature hinders trust.
Zafar et al. ^10^	- Preserves user data privacy by avoiding centralized storage. - Enables collaborative model training across distributed nodes. - Improves robustness with hybrid architectures.	- Communication overhead during model updates. - Sensitive to data heterogeneity across devices. - High implementation complexity.
Huang et al. ^11^	- Combines short-term and long-term data features for better accuracy. - Effective in addressing temporal dependencies in theft detection. - Reduces false positives.	- Increased complexity due to feature fusion. - Requires high-quality time-series data. - Computationally intensive.
Shehzad et al. ^12^	- Adaptive to diverse theft detection environments. - Enhances generalization across datasets. - Supports transfer learning for quick deployment.	- High computational and storage requirements. - Design and implementation complexity. - Limited interpretability.
Kawoosa et al. ^13^	- High predictive accuracy by combining multiple models. - Robust to noise and missing data. - Easy to interpret compared to deep learning.	- Requires extensive feature engineering. - Less scalable for large AMI networks. - Computational cost increases with ensemble size.
Yan et al. ^14^	- High accuracy and speed. - Handles imbalanced datasets well. - Provides feature importance for interpretability.	- Memory-intensive for large datasets. - Requires manual parameter tuning for optimal performance. - Less effective for highly dynamic patterns.
Jindal et al. ^15^	- Computationally efficient and interpretable. - Suitable for small to medium-sized datasets. - Easy to implement in real-world scenarios.	- Limited scalability for large datasets. - Lower accuracy compared to deep learning models. - Struggles with non-linear and complex patterns.
Mohammad et al. ^16^	- Combines multiple models for better performance. - Effective in reducing false positives. - Provides interpretable decision support.	- High computational cost due to multiple model training. - Relies on high-quality data preprocessing. - Sensitive to overfitting with complex ensembles.

**Table 2:** List of related works algorithms, data sources, and limitations.

Refs.	Methodology	Dataset	Limitation	Result (%)
El-Toukhy et al.^6^	REINFORCEMENT LEARNING + DNN	ISET	Inefficient adaptation Lack of exploration Generalization problem	ACC.: 97.3
Emadaleslami et al.^7^	CNN + DNN	ISET	More computation time might be needed	ACC.: 98.1
Lepolesa et al.^8^	DNN	^55^	–	ACC.: 91.8
Pamir et al.^9^	RNN	SGCC	More computation time	ACC.: 92.3
Zafar et al.^10^	DNN + CNN + LSTM	Open energy data initiative^56^	costly due to the installation of devices and sensors	ACC.: 97.8
Huang et al.^11^	CNN + LSTM	SGCC	–	ACC.: 94.7
Shehzad et al.^12^	ML	PREC	Class imbalance problem	ROC-AUC: 0.910 PR-AUC: 0.988
Kawoosa et al.^13^	PCA + ML	SGCC	–	ACC.: 94
Yan et al.^14^, 2021	XGBoost	ISET	Limited results due to analyses electricity consumption data alone	Detective rate (DR): 95.2
Jindal et al.^15^	Decision Tree + SVM	^15^	Require more features	ACC.: 92.50
Mohammad et al.^16^	ML + DNN	SGCC	Small dataset Computational complexity and cost Can not detect real-time electricity.	ACC.: 88–94.70%

## Materials and methodologies

### Dataset description

Electricity theft poses a significant challenge for utility providers worldwide, resulting in revenue losses and safety hazards. Deep learning (DL), a subset of artificial intelligence, has emerged as a promising tool to combat this issue. Through the integration of DL technologies, utility providers can improve their ability to detect and deter electricity theft, ultimately promoting a more reliable and secure energy infrastructure. The dataset “electricity theft detection,”^39^ released by the State Grid Corporation of China (SGCC), comprises 1037 columns and 42,372 rows representing electric consumption data from January 1st, 2014, to October 30th, 2016. The first column in the SGCC dataset contains alphanumeric consumer IDs, while columns 2 through 1036 contain daily EC values. The final column, labelled “flag,” indicates theft occurrences with values of 0 or 1.

**Fig. 1:**
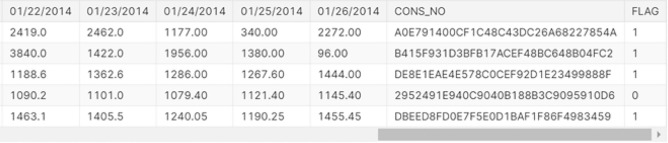
A sample of the dataset^39^.

This dataset encompasses a wide array of information, including historical and real-time data on energy consumption, grid infrastructure, integration of renewable energy sources, and grid performance. It plays a pivotal role in enhancing the reliability and efficiency of power distribution networks by facilitating tasks such as demand prediction, grid monitoring, and issue identification. This dataset includes missing values and imbalanced data for theft and honour. A sample of the used dataset is shown in Fig. 1.

### Dataset pre-processing

Most datasets have problems such as missing data and imbalance. Fill missing data is the first step in our dataset treatment. Insufficient data may skew the outcomes of ML models and/or diminish their accuracy. There are different causes for missing data and also there are many ways to fill the missed data.

To address missing values, start by thoroughly examining the entire dataset to identify and locate any absent values. Many techniques exist for substituting missing values such as using an arbitrary value, replacing with the mean, employing the mode, and substituting with the previous value. In this paper, we replace the missing values with mode values.

**Fig. 2:**
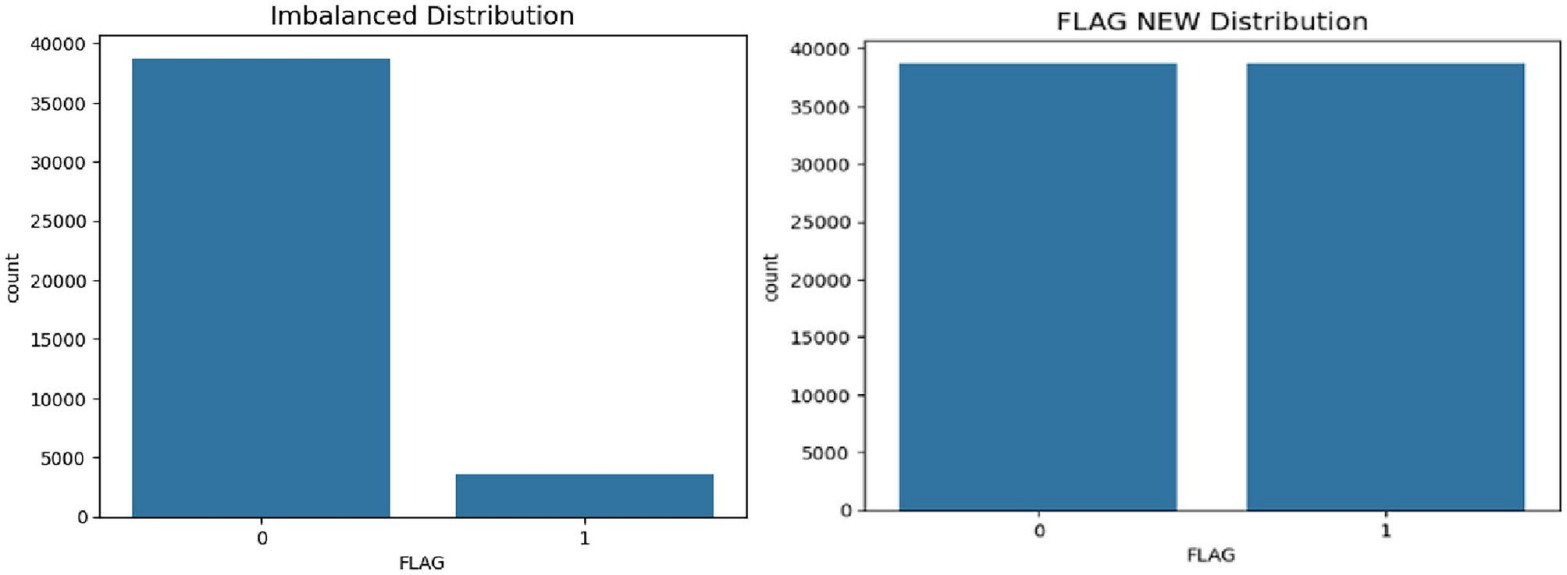
The effect of use LoRAS balance technique: on the left imbalanced dataset while on the right balanced dataset.

The second set in the dataset pre-processing is solving the imbalance of data. Imbalanced datasets are common across various fields where ML is applied, such as business, finance, banking, and bio-medical science. SGCC dataset was an imbalanced data and also need some preprocessing to satisfy the used with deep learning models. The missing values problem was solved by using the mode operation to fill the missing data. Min-Max scaler was used for normalization to scale the range in [− 1, 1]. Selecting the target range depends on the nature of the data. Then, the problem of imbalanced data was solved by using Localized Random Affine Shadow sampling (LoRAS)^64^, which involves approximating the manifold locally by generating random convex combinations of noisy minority class data points. Our oversampling strategy, LoRAS, aims to enhance the precision-recall balance (F1-Score) and the class-wise average accuracy (Balanced accuracy) of the ML models utilized. The F1-Score evaluates the effectiveness of the classification model in handling minority class classification, while Balanced accuracy provides insight into how both majority and minority classes were managed by the classification model. Therefore, combining these two metrics offers a comprehensive assessment of classifier performance on a dataset. Figure 2 shows SACC dataset distribution before and after the use of LoRAS data augmentation technique. The dataset is publicly used as reference data for energy theft detection, making it the best reference to show how accurate our model is, comparing it to a state-of-the-art model.

### Methodologies

The objective of this paper was to develop a deep learning model for classifying electricity theft. The prediction task is approached as a classification problem, where the model output is binary: (0) indicates a customer involved in theft, while (1) indicates an honest customer. This section outlines the architecture of the proposed models designed for electricity theft classification.

**Fig. 3:**
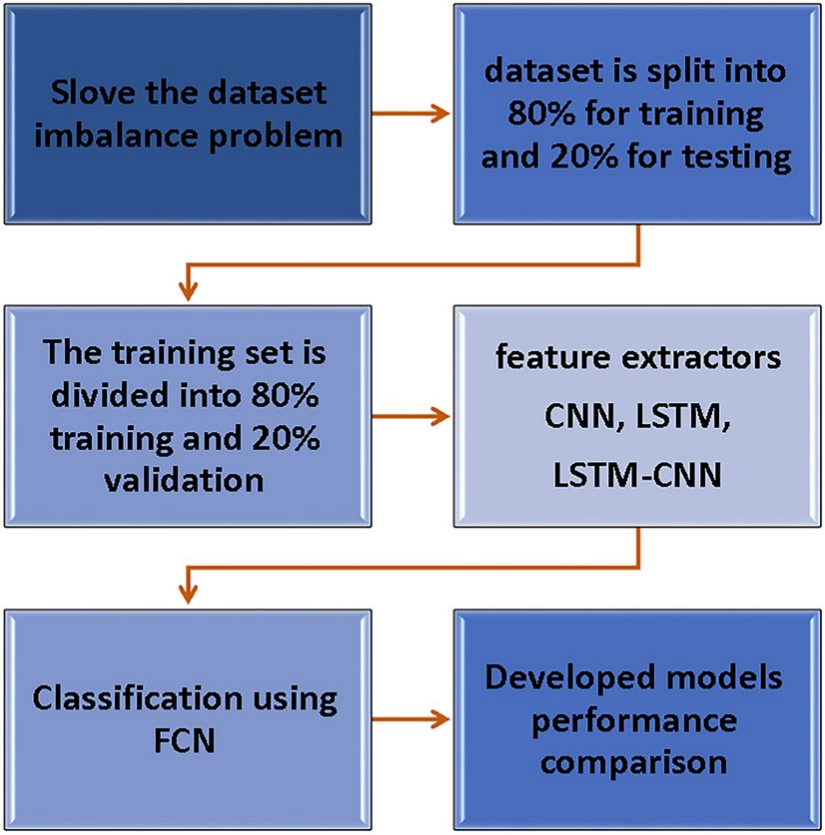
Block diagram of the methodology used in this study.

Figure 3 illustrates the methodology employed in this study. Initially, the dataset’s class imbalance between theft and honest customers is addressed using LoRAS. Subsequently, the dataset is split into 80% for training and 20% for testing. The training set is further divided into 80% for training and 20% for validation. Different deep learning techniques (CNN, LSTM) serve as feature extractors, followed by fully connected neural networks as classifiers. Finally, our findings are compared to state-of-the-art studies with the same objective on the same dataset. The pseudocode for the proposed algorithm shown in Table 2.

**Algorithm 1:**
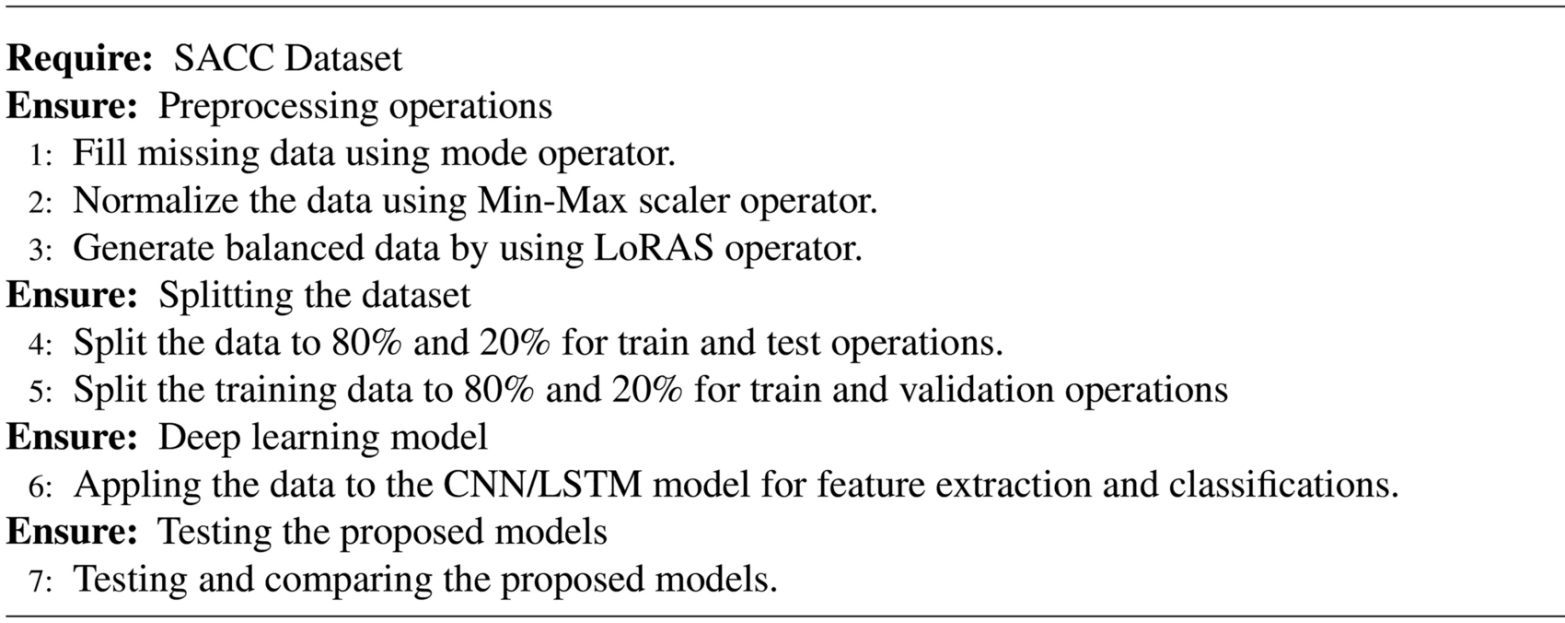
Pseudocode of the proposed model.

#### Deep learning techniques

Deep learning, a subset of ML, involves neural networks consisting of three or more layers. While these networks attempt to replicate the functionality of the human brain, they cannot currently fully emulate its capabilities, particularly in terms of learning from extensive datasets. Although even a single-layer neural network can provide approximate predictions, additional hidden layers are often employed to enhance accuracy through tuning and refinement. Among the well-known deep learning models are convolutional neural networks (CNNs) and recurrent neural networks (RNNs).

#### Convolutional neural networks (CNN)

In recent years, the utilization of deep learning in clinical diagnosis and medical imaging has experienced rapid growth, particularly with convolutional neural networks (CNNs)^65^. CNNs are a specialized form of multi-layer neural networks specifically designed to directly recognize visual patterns from pixel images with minimal preprocessing. Their advantages include the capacity to extract more meaningful features from data and images compared to handcrafted features. Researchers have proposed various CNN-based deep networks for tasks such as image classification^66^, segmentation^67^, object detection, and localization in computer vision^68^. Beyond traditional computer vision applications, CNNs have demonstrated remarkable effectiveness in addressing medical challenges such as breast cancer detection, brain tumour segmentation, Alzheimer’s disease diagnosis^69^, and skin lesion classification^70^. CNN stands out as the primary tool for feature extraction owing to its exceptional performance. Its architecture comprises three main components: input, output, and one or more hidden layers. These hidden layers encompass various elements including convolution, normalization, pooling, and concatenation. Specifically, the CNN is structured with an input layer, an output layer, and one or more hidden layers situated in between, each equipped with components facilitating convolution and normalization processes. CNN-based models have shown strong and reliable performance across a range of research applications.

#### LSTM

Recurrent neural networks (RNNs) represent another category of deep learning techniques primarily employed for prediction tasks. They offer significant advantages in achieving robust modelling and prediction performance. Among the notable RNN variants, long short-term memory (LSTM) stands out, particularly for handling large neural networks^71^. LSTM’s key advantage lies in its capability to effectively model both short and long-term memory, overcoming the vanishing gradient issue encountered in traditional RNNs when training on lengthy sequences. These two techniques constitute the primary pillars of deep learning methodologies. Their potential inspired us to devise an architecture combining convolutional neural networks (CNNs) and LSTMs, leveraging the strengths of both approaches to facilitate feature extraction and image classification effectively^72^.

#### Proposed models

**Fig. 4:**
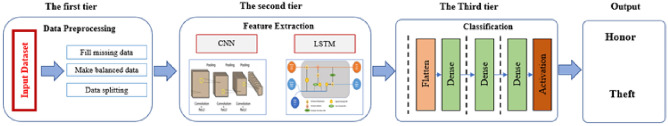
Electricity theft detection DL framework.

As observed in the literature, numerous deep learning models have been introduced for addressing ETD from data. These models incorporate various performance metrics to assess their validity. However, a significant ongoing challenge is to identify a powerful, suitable, and efficient model that meets all performance criteria. The objectives of our study encompass (i) proposing a deep learning framework for electricity theft classification, employing an innovative feature extraction method, and (ii) evaluating this framework by comparing it with recently introduced models. Our developed deep learning framework, depicted in Fig. 4, comprises three main tiers. The first tier focuses on data pre-processing tasks such as handling missing data, data partitioning, and addressing data imbalance issues. The second tier is dedicated to feature extraction by using three different deep learning techniques: CNN, LSTM, and CNN-LSTM; each has the same inputs, and the output of this tier is the concatenation of the generated features by the three techniques. Finally, the third tier is responsible for classification decisions.

#### The proposed architectures

The proposed models use CNN and LSTM for feature extraction, as well as CNN for the classification of the detection of theft of electricity. The design and implementation of the proposed approach comprise three steps: data preprocessing, feature extraction, and classification. The following are the stages of the feature extraction and classification architecture. In our paper, two different deep learning model were introduced to solve the electricity theft problem. These models use two techniques: CNN and LSTM. The architecture of these models presented in next sections.

#### CNN model

**Fig. 5:**
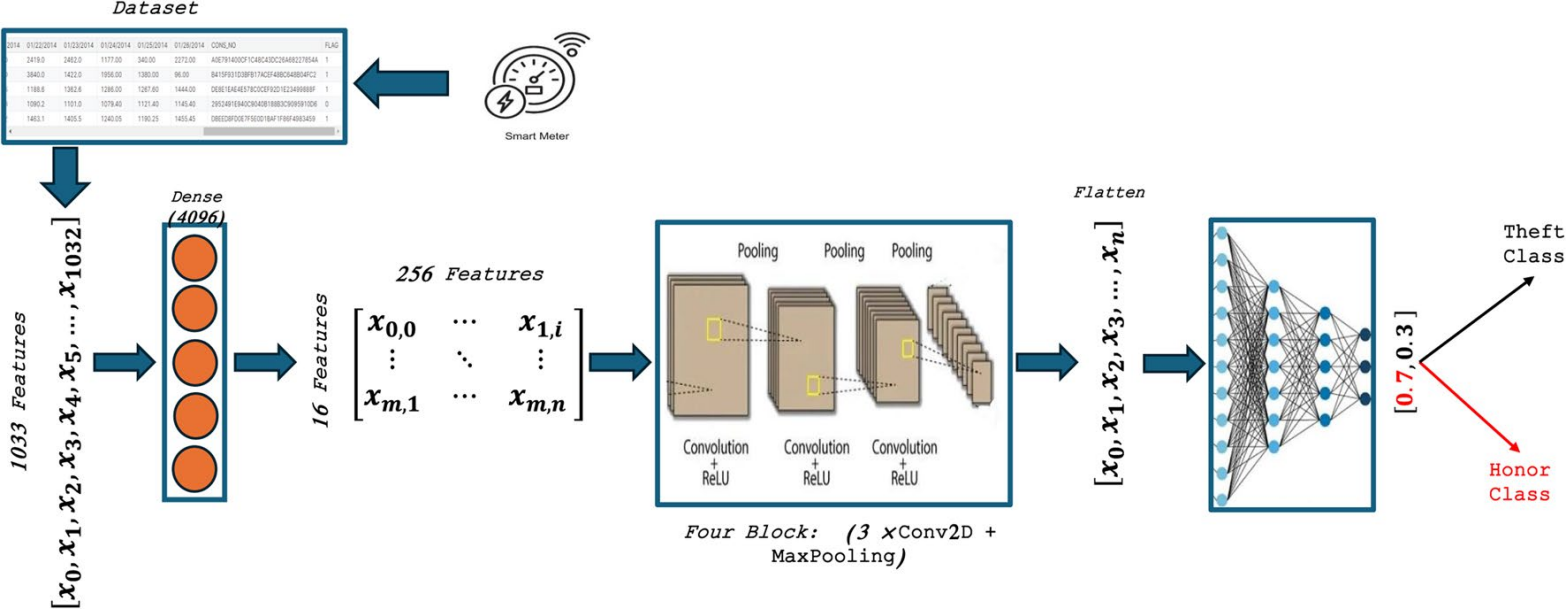
Proposed CNN architecture.

Deep neural networks integrate convolutional neural networks (CNNs) for feature extraction and classification to detect electricity theft. Figure 5 showshe CNN model in this study, which consists of input, feature extraction, and classification layers. The input layer consists of 1033 units. A dense layer of 4096 neurons receives the data to transform it into a 2-dimensional format. We use the feature extraction section, which is made up of 5 CNN blocks, each with 3 convolutional layers with different filter numbers and rectified linear unit (ReLU) layers. This is because CNN is very good at extracting features for image classification^66^. The five blocks have 128, 64, 64, 32, and 16. Optionally, each block may include maximum pooling layers, as depicted in Fig. 5. The feature extraction section’s output then feeds into a flattened layer, transforming the data shape into a 1-dimensional vector, a format suitable for the classification dense layer. This architecture utilizes two dense layers, each containing 128 and 64 neurons, along with dropout layers. The dense layer with sigmoid activation generates the final output.

#### LSTM model

**Fig. 6:**
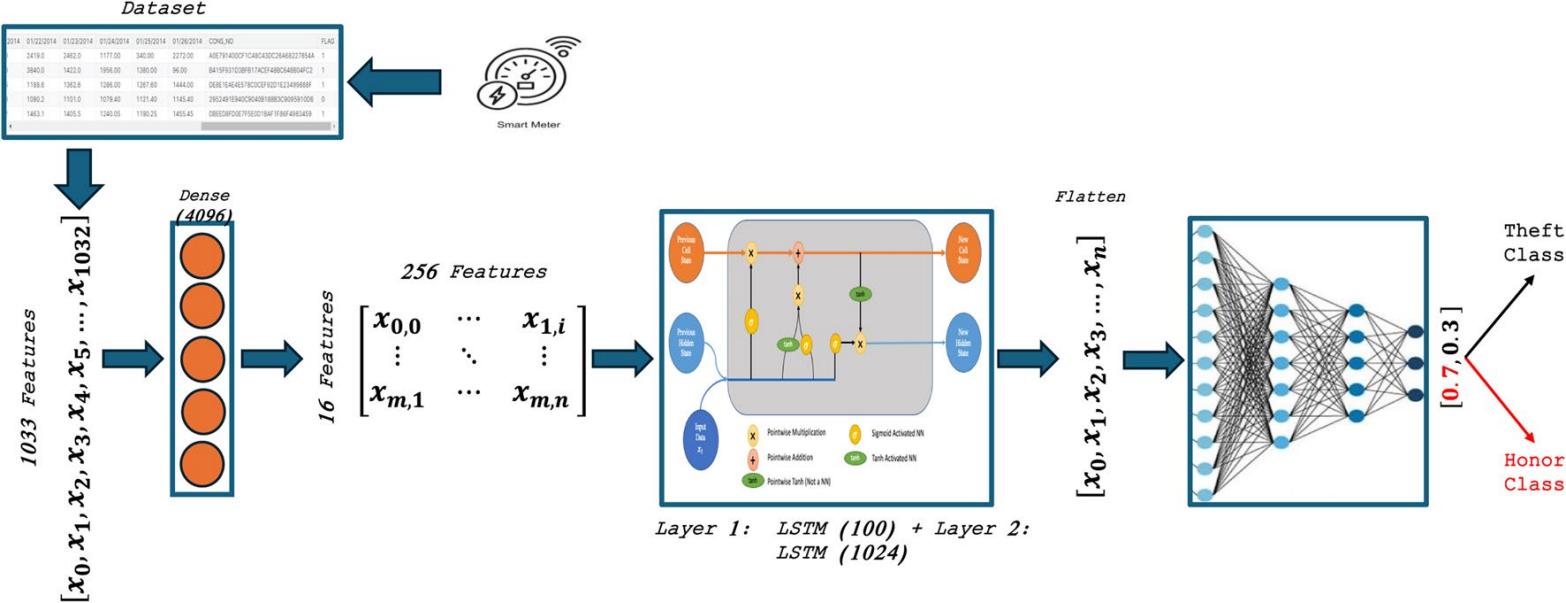
Proposed LSTM architecture.

LSTM, a type of recurrent neural network (RNN) architecture, excels at modelling time series data. Figure 6 illustrates the proposed LSTM model in this study. This architecture applies a dense layer before the LSTM to preprocess the input for optimal LSTM performance. The classification stage uses a flattened layer and two sets of dense layers, each with 100 and 1024 neurons, for classification. For classification, we add dropout layers with a factor of 0.3 and, finally, a dense output layer with a sigmoid activation function.

### Proposed models evaluation

The experiments utilize a publicly available dataset^39^ comprising two distinct classes: one representing electricity theft and the other representing legitimate customers. Each sample is split, with 80% allocated for training and 20% for validation. The training time of the proposed model around 200 second per epoch and the training for each model take about 20 epochs, so the training time for each model is 4000 second. The models are implemented in Python 3 using the Keras framework. The resource requirements are Google Colab with GPU acceleration and an Intel(R) Xeon(R) CPU @ 2.20GHz processor with 13 GB RAM. The classification process involves applying the trained deep-learning models to the validation dataset. In the case of the fake-ensemble model, test samples are distributed to individual models, each producing a prediction. Subsequently, a majority voting technique is applied to the results of all base classifiers to generate the final prediction. The code will be made available on GitHub. Using a dataset for energy theft detection highlights concerns about privacy implications and the need for protections to secure sensitive information^73^. Protecting the privacy of sensitive user data must be considered for many reasons, such as Monitoring power usage data, which offers information about daily activities and behaviours, including the customer’s presence at home or departure and daily life patterns^73,74^. Encryption may be used to ensure and safeguard customer identification and solve these issues. Furthermore, minimal data gathering will minimize the chances of abuse^75^.

The proposed model offers privacy for customers because it will apply to SMs and will not allow anyone to read customer data. The only ones with the right to access this data are the customer and the electricity service provider.

The trained deep learning models perform classification on the test dataset. In the proposed fake-ensemble model, which incorporates CNN, LSTM, and LSTM-CNN for feature extraction, the features produced by these models are concatenated and passed through fully connected neural networks serving as the classification model to determine the final classification decision. The framework takes advantage of a deep learning model to learn from raw data and filter out noise, focusing on essential data^76^. Moreover, the first step in the first tier of the framework is used to overcome the missing data due to noise or a problem of transferring the data, using bilinear interpolation by estimating the missing values.

#### Performance metrics

To assess the models’ performance, four commonly used performance evaluation metrics are employed: true negatives (TN), true positives (TP), false negatives (FN), and false positives (FP). Additionally, four evaluation metrics are utilized: recall, precision, accuracy, and F1-score, as defined in Eqs. (1–9). Recall, also known as sensitivity or true positive rate, measures the proportion of positive instances correctly predicted out of all actual positive instances. Precision, or positive predictive value, gauges the proportion of correctly predicted positive instances out of all instances predicted as positive. Accuracy represents the ratio of correctly predicted instances to the total number of instances. F1-score integrates Precision and Recall into a single metric using their harmonic mean. Specificity, on the other hand, denotes the proportion of correctly predicted negative instances out of all actual negative instances^66^.

1 $$\begin{aligned} Sensitivity (Sens.)= & \frac{Tp}{Tp+Fn} \end{aligned}$$

2 $$\begin{aligned} Precision (Pre.)= & \frac{Tp}{Tp+Fp}\end{aligned}$$

3 $$\begin{aligned} Accuracy (Acc.)= & \frac{Tp+Tn}{Tp+Tn+Fp+Fn}\end{aligned}$$

4 $$\begin{aligned} F1-score= & \frac{2*Precision*Sensitivity}{Precision+Sensitivity}\end{aligned}$$

5 $$\begin{aligned} Negative Predictive Value (NPV)= & \frac{Tn}{Tn+Fn}\end{aligned}$$

6 $$\begin{aligned} False Positive Rate (FPR)= & \frac{Fp}{Fp+Tn}\end{aligned}$$

7 $$\begin{aligned} False Discovery Rate (FDR)= & \frac{Fp}{Fp+Tp}\end{aligned}$$

8 $$\begin{aligned} False Negative Rate (FNR)= & \frac{Fn}{Fn+Tn}\end{aligned}$$

9 $$\begin{aligned} Matthews\,Correlation\,Coefficient\,(MCC)= & ((T_P\times T_N ) -(F_P\times F_N))\\&/\sqrt{((T_P+F_P)\times (T_P+F_N)\times (T_n+F_P)\times (T_N+T_n))} \end{aligned}$$

where, TP represents true positives, indicating correctly classified positive instances. TN stands for true negatives, representing correctly classified negative instances. FP refers to false positives, indicating incorrectly classified positive instances. Lastly, FN denotes false negatives, representing incorrectly classified negative instances.

#### Experimental results and comparative analysis

We define the performance metrics used, the proposed system’s implementation details, and the experiments conducted for evaluation. Several prominent methods for performance comparison are considered, along with popular evaluation parameters such as accuracy, precision, recall, and F1 score. The prediction performance evaluation begins with a statistical analysis of the predicted classes. We then examine the training and prediction characteristics, which include loss, AUC, precision, recall, and accuracy vs. epoch curves and confusion matrices. Finally, we compare the validation accuracy of our proposed model with other prominent models on the integrated dataset.

#### Training metrics per epoch performance

**Fig. 7:**
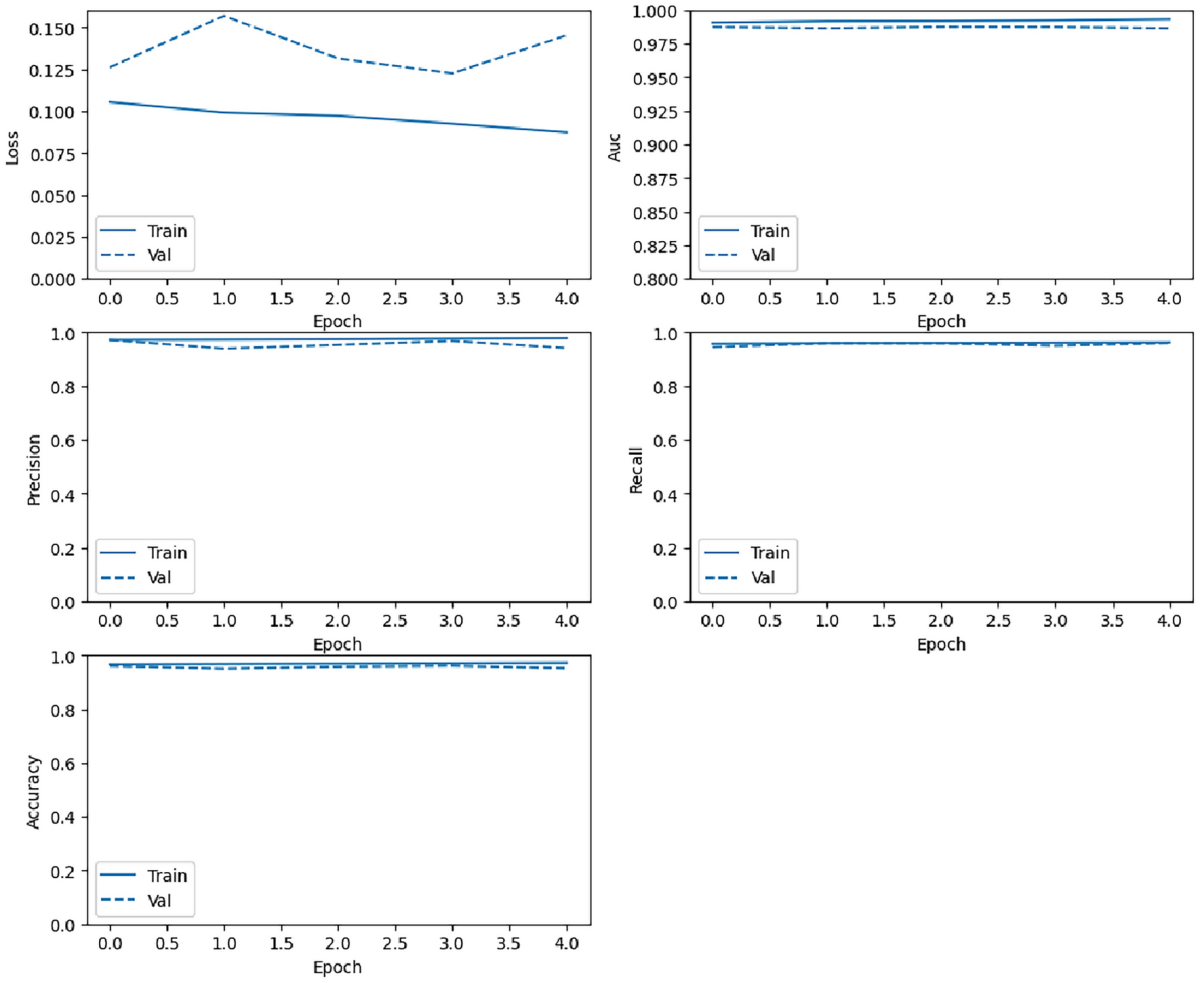
Training loss, AUC, precision, recall, and accuracy changes with epoch (from top to bottom, left to right) for the CNN model.

**Fig. 8:**
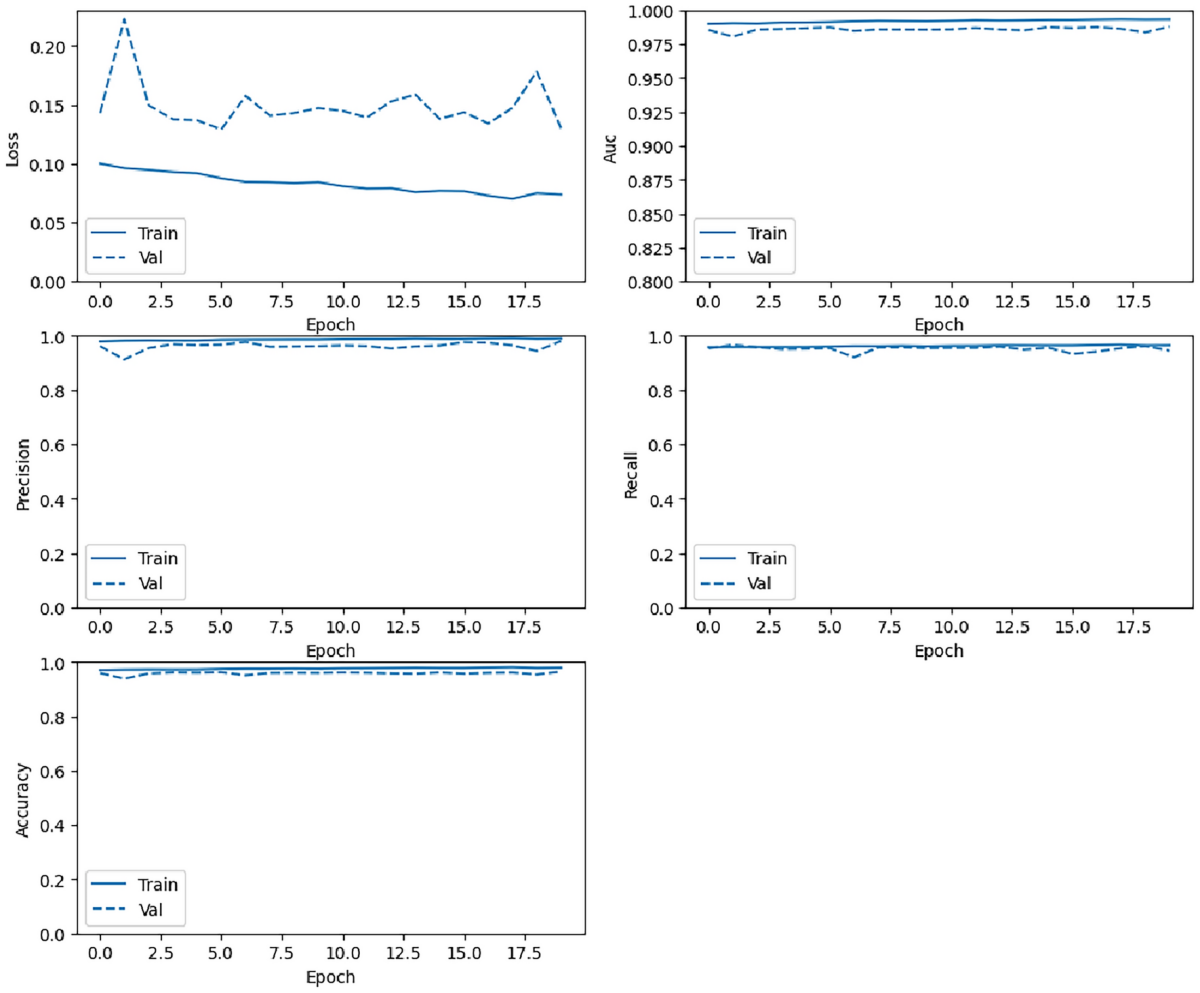
Training loss, AUC, precision, recall, and accuracy changes with epoch (from top to bottom, left to right) for the LSTM model.

For a DL model to be considered a perfect match, the training and validation properties must exhibit a high degree of alignment. We usually assess this association by comparing several measurements to epoch curves. Hence, in a well-crafted model, the accuracy should steadily increase with each epoch, with minimal variations, whereas the loss of each epoch should reflect an opposite pattern. Both measures should have a comparable trend throughout the training and validation stages. The features of our two DCNN models LSTM and CNN are shown by the loss, AUC, precision, recall, and accuracy vs. epoch curves, as depicted in Figs. 7 and 8. This became particularly evident throughout the advanced phases of training when the loss began to increase. However, both the cumulative loss and accuracy measures indicate a significant resemblance between the validation and training outcomes. This suggests that the models possess the ability to successfully apply their learned knowledge to new data and have not encountered substantial issues of overfitting when the models excessively adapt to the training data and perform poorly on new data.

#### Confusion matrix

A helpful method for summarizing a classification’s performance is a confusion matrix. Relying just on prediction accuracy might be misleading when the dataset has more than two classes or when the distributions of the classes are unbalanced. We may get a better understanding of the classification model’s accomplishments and shortcomings by computing a confusion matrix. We anticipate that a deep learning model that works well would have values in the confusion matrix’s diagonal cells that are near to one, signifying precise predictions for every associated class.

**Fig. 9:**
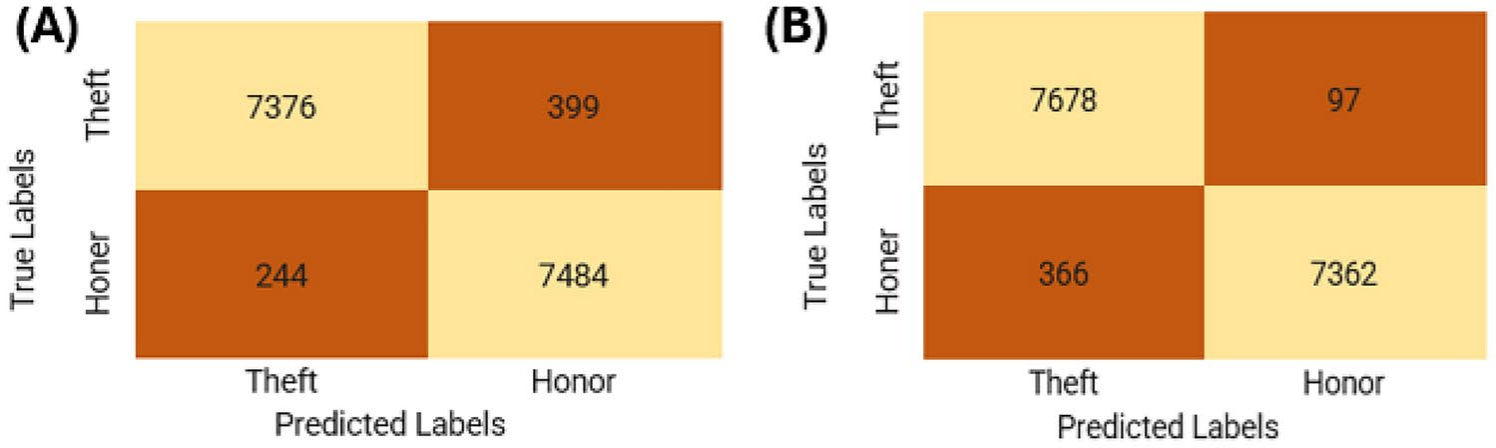
Training loss, AUC, precision, recall, and accuracy changes with epoch (from top to bottom, left to right) for the LSTM model.

The confusion matrices for our classification models are illustrated in Fig. 9. The LSTM model outperformed the other two models under evaluation, misclassifying the theft class just 97 times. In the test dataset, this model misclassified just 366 items, indicating its excellent overall accuracy.

### Performance comparison

We are presently evaluating the classification capabilities of many binary-class ETD classification models to determine which one performs the best. The dataset, the prediction performance measures, and the brief technical specifications for each model are included in this comparison. The outcomes are shown in Table 3. We assess the predicted performance of the models under consideration using the total test accuracy percentile as a criterion. Significant differences exist between the test accuracy results for each model. This variance may be explained by variations in each model’s training strategies on the dataset or in its model architectures.

**Table 3:** Different performance metrics for CNN and LSTM models.

Model	Acc.	F1 Score	MCC	Sens.	Spec.	Pre.	NPV	FPR	FDR	FNR
CNN	95.85	95.82	91.72	96.80	94.94	94.87	96.84	5.06	5.13	3.20
LSTM	97.01	97.07	94.08	95.45	98.70	98.75	95.26	1.3	1.25	4.55

**Fig. 10:**
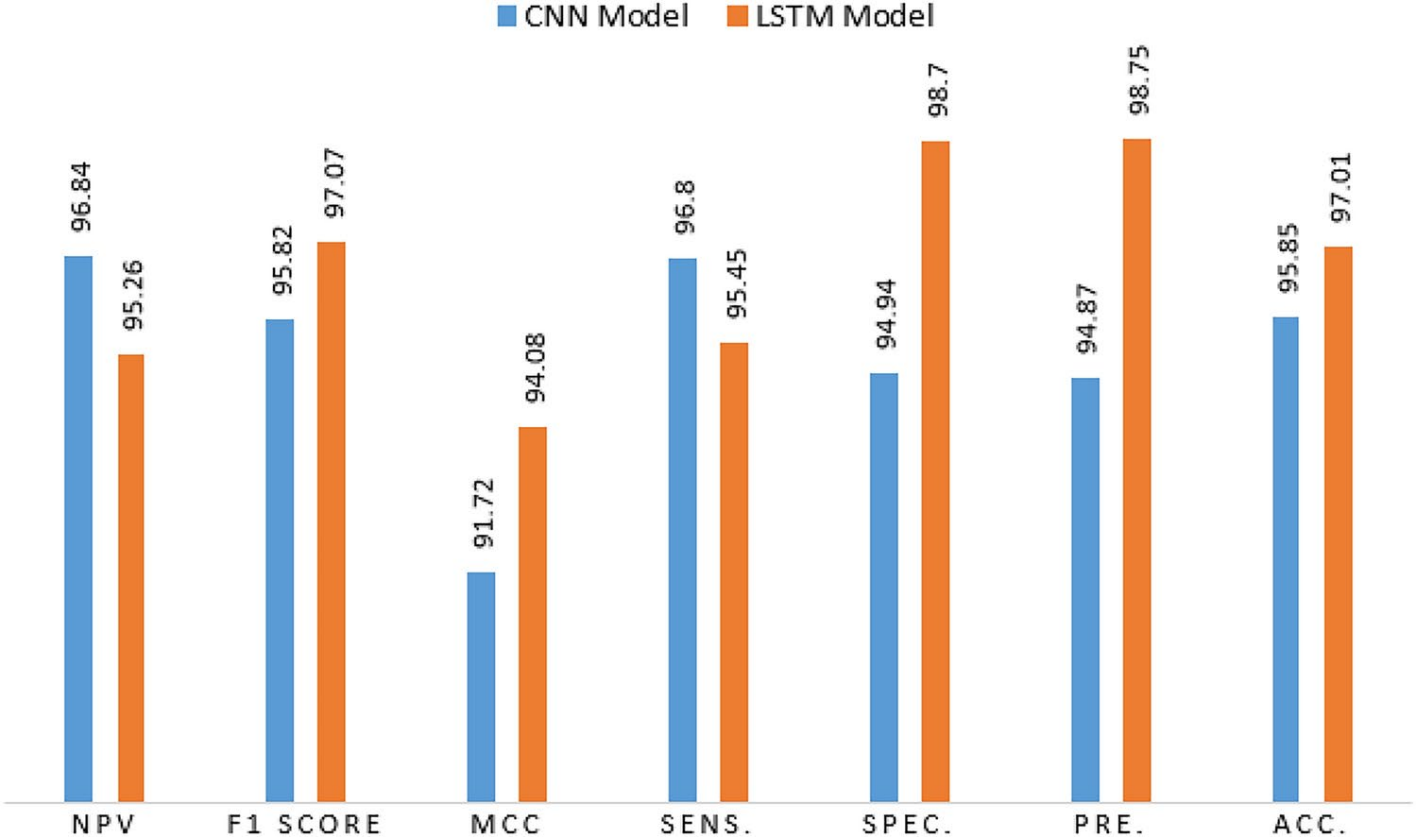
The performance metrics for CNN and LSTM models.

**Fig. 11:**
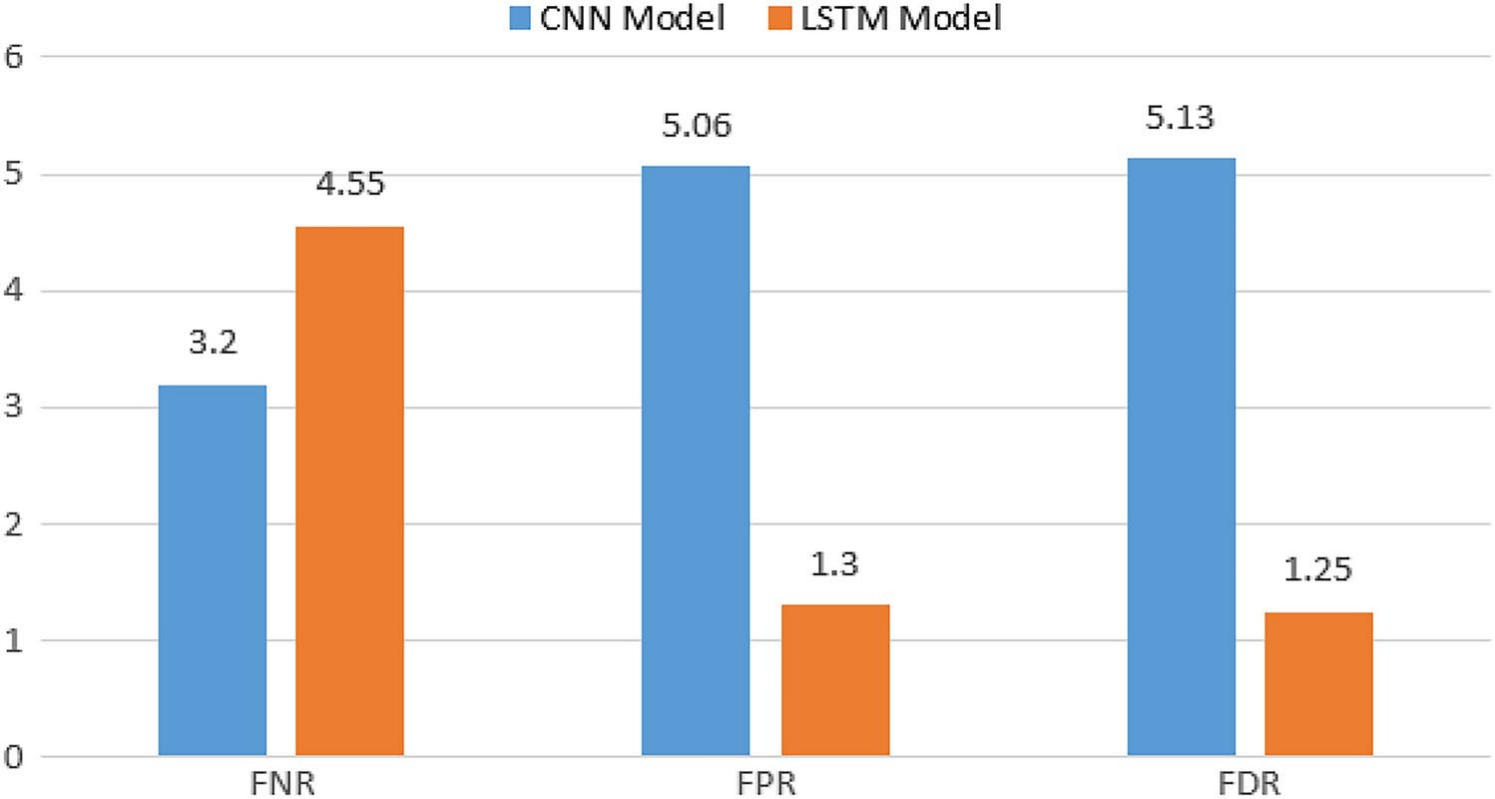
The FNR, FPR, and FDR performance metrics for CNN and LSTM models.

To achieve a highly accurate framework for classifying user behaviour, two models based on deep learning were tested (the CNN and LSTM models). Different performance metrics are tested to select the best classification model. The proposed LSTM model achieves a high accuracy score of 97.01%, outperforming the CNN model with a score of 95.85%. Moreover, other performance metrics also demonstrate a high rate of success. The sensitivity focuses primarily on true positive results, achieving a score of 95.45% for LSTM and 96.8 for CNN. Specificity focuses on actual negatives correctly identified by the model, achieving 98.7% and 94.94% for LSTM and CNN, respectively. The precision measure of the proportion of correct positive predictions achieves 98.75% for LSTM and 94.87% for CNN. The MCC measures the correlation coefficient between the true and predicted binary classifications when the MCC is nearest to 100%, which is the mean a model has high correlation. The LSTM has a correlation coefficient of 94.08%, whereas the CNN achieved a result of 91.72%. NPV, which is responsible for the percentage of negative predictions that are actually correct, is also measured, and the LSTM model achieves 95.26%. Figure 10 illustrates the performance metrics for CNN and LSTM models. Other metrics, such as FPR, FDR, and FNR, were also tested. The LSTM has 1.3, 1.25, and 4.55, respectively. The CNN model achieves 5.06, 5.13, and 3.2. Figure 11 shows the comparison graph for both models.

#### Comparison to state-of-the-art methods

We are contrasting our suggestion with the work of Pamir et al.^9^, who achieved an accuracy of 92.3% by developing a Long Short-Term Memory (LSTM) model. In addition, Huang et al.^11^ combine the Convolutional Neural Network (CNN) and Long Short-Term Memory (LSTM) models using various kinds of features, resulting in a remarkable accuracy of 94.7%. We conducted a comparison between our work and the ML algorithm created by Kawoosa et al.^13^ and Mohammad et al.^16^. Our findings indicate that LSTM achieved a higher accuracy rate than the aforementioned algorithms. We conducted this work using the same dataset. Using LoRAS as data augmentation with the LSTM model, we obtained the best accuracy of 97%. Table 4 compares the accuracy of the proposed LSTM model with some related studies. The suggested model surpasses other deep learning techniques.

**Table 4:** Comparison of performance metrics.

Ref.	Methodology	Result
Pamir et al.^9^	RNN	ACC.: 92.3%
Kawoosa et al.^13^	PCA + ML	ACC.: 94%
Mohammad et al.^16^	ML + DNN	ACC.: 94.70%
Huang et al.^11^	CNN + RNN	ACC.: 94.7%
Proposed model 1	CNN	ACC.: 95.85%
Proposed model 2	LSTM	ACC.: 97%

## Conclusion

This research aims to improve the precision of theft detection for energy users using a data-driven methodology. Using deep learning models, we analyze power usage characteristics at various time scales and exploit the benefits of feature extraction. Therefore, we provide an innovative theft detection approach that relies on CNN and LSTM. We assessed the model’s performance using established performance criteria such as accuracy, precision, F1-score, and recall. Our observation revealed that LSTM classification surpassed CNN and other state-of-arts methods. The classifier demonstrated a 97% accuracy rate when assessed on a testing set. When training a model, extracting a balanced dataset from the whole training set restricts the model’s biases. It enhances its ability to evaluate the performance of the proposed model on an imbalanced dataset. Our goal is to expand our methodology to identify real-time power theft instances. We assessed the efficacy of this approach by analyzing SGCC consumers’ consumption habits. Additionally, we can verify its suitability across several regions by comparing it to datasets from different locales.

## Data Availability

The datasets used and analyzed during the current study are available from the corresponding author on reasonable request.
